# Impact of ketogenic diet and ketone diester supplementation on body weight, blood glucose, and ketones in Sprague Dawley rats fed over two weeks

**DOI:** 10.1016/j.fochms.2021.100029

**Published:** 2021-06-10

**Authors:** LT Claire M. Modica, Krystal Flores-Felix, LT John D. Casachahua, Paul Asquith, Anna Tschiffely, Stephanie Ciarlone, Stephen T. Ahlers

**Affiliations:** a Naval Medical Research Center, Silver Spring, MD, United States; b Universidad Ana G. Méndez, Gurabo Campus, PR, United States; c Hazel Green High School, Hazel Green, AL, United States; d Huntingdon College, Montgomery, AL, United States; e The Henry M. Jackson Foundation for the Advancement of Military Medicine, Inc., Bethesda, MD, United States

**Keywords:** Ketogenesis, Ketosis, Ketogenic, Ketone ester, Ketone diester, Ketone bodies

## Abstract

Ketogenic diets consist of low carbohydrate/high fat, shifting energy reliance from glucose to ketone bodies. Ketone diester supplement to a standard diet (ketone ester) increases ketone bodies by adding a substance without altering other consumed foods. We evaluated weight, glucose, and ketone concentrations in rats fed ketogenic diet and ketone ester feeds. We hypothesized that these feeds would increase ketones and decrease glucose and weight. We tested 16 male and 16 female Sprague Dawley rats randomly assigned to standard diet, ketogenic diet, or ketone ester for two weeks. Weight and blood glucose and ketones were measured daily. Group means were compared by analysis of variance. Ketogenic diet and ketone ester both increased ketones and decreased weight compared to standard diet (p < 0.001). Glucose decreased only in ketogenic diet (p = 0.010), driven by a decrease from higher starting concentrations observed in standard diet males. Sex interacted with weight, with male gains impacted more by both ketogenic diet and ketone ester than female gains. Ketogenic diet had a larger effect size than ketone ester with regard to increased ketones and decreased weight. Ketogenic diet glucose significantly decreased over time because standard diet concentrations in males were high prior to initializing ketogenic diet. This suggests sex differences in energy substrate utilization. Ketogenic diet ketones peaked at 72 h then decreased to near basal levels at about 10 days, suggesting “fat adaption.” While this work is part of a larger project examining blast exposure, these results are relevant to any military forces considering ketone-increasing foods.

## Highlights

• Ketogenic diet influenced ketones, weight, and glucose in rats.
• Ketone diester supplement (20% by weight) was similar to ketogenic diet, but its effects on ketones and weight was smaller and it did not affect glucose.
• Changeover from standard diet to ketogenic diet resulted in sex-specific glucose changes.

## 1 Introduction

Resilience in the face of injury may rely on molecular energy availability. While inactive neurons default to lactate use (Pellerin & Magistretti, 1994), actively firing excitatory neurons require glucose to sustain glutamate release (Bak, Schousboe, Sonnewald, & Waagepetersen, 2006). Because head trauma is associated with glucose metabolism depression (Giza & Hovda, 2014) and reduced availability of ATP (Lifshitz, Sullivan, Hovda, Wieloch, & McIntosh, 2004), we planned to develop a ketone metabolism blast injury model. In preparation, we explored two ketone-producing dietary conditions.

When oxygen availability is limited in traditional glucose metabolism, anaerobic glycolysis leads to an increased production of lactate, resulting in reduced ATP output downstream. In ketogenesis, the glycolytic step is not crucial to ATP production, so anaerobic glycolysis is no longer a concern. In the ketogenic diet, fatty acids are largely oxidized into ketone bodies in the mitochondria of the liver (ketogenesis), then transported to the mitochondria of target tissues where they are turned into acetyl-CoA. Acetyl-CoA is then fed into the citric acid cycle, which then feeds downstream oxidative phosphorylation and the electron transport chain, ultimately resulting in synthesis of ATP (Vidali et al., 2015). Ketone diester supplementation to a standard diet (ketone ester) is thought to work similarly. The ketone diester, R,S-1,3-butanediol diacetoacetate, is metabolized into three ketone bodies (beta-hydroxybutyrate, acetoacetate, and their spontaneous breakdown product, acetone). These ketone bodies then utilize the same processes ramped up by the ketogenic diet, sustaining ketogenesis (Pilla, 2017).

This project examined ketogenic diet and ketone ester, fed ad libitum. Blood glucose, blood ketones, and body weight under these diet conditions were compared to standard rodent laboratory chow (standard diet) over two weeks in male and female Sprague Dawley rats.

## 2 Materials and methods

The protocol was approved by the Walter Reed Army Institute of Research/Naval Medical Research Center Institutional Animal Care and Use Committee, and all institutional health and safety procedures were complied with. Standard diet feed was Prolab isopro RMH 3000 (5P76) (PicoLab, Fort Worth, TX, USA); ketogenic diet feed was purchased off-the-shelf (#F3666, Bio-Serv, Flemington, NJ, USA); ketone ester feed was custom-made (Dyets, Bethlehem, PA, USA) and consisted of Prolab isopro RMH 3000 (5P76), 20% ketone diester by weight (as described (Kesl et al., 2016)), and gifted from Disruptive Nutrition, LLC, Durham, NC, USA), 4% peanut butter, and 1% saccharin.

For the initial comparison of ketogenic diet to standard diet, eight male and eight female Sprague Dawley rats were randomly assigned to either diet for two weeks by feeding the list of conditions through a random number generator function in SPSS (IBM, Armonk, NY, USA). Body weight, blood glucose levels, and blood ketone levels were measured daily. Blood was sampled at the tail vein daily and analyzed by Precision Blood Glucose/Ketone Meter (Abbott Laboratories, Abbott Park, IL, USA). Observations of the ketogenic diet condition during the initial comparison between ketogenic diet and standard diet were confirmed by a second ketogenic diet sample in measurements that were only taken twice: prior to diet changeover and once more two weeks later. A third cohort of eight male and eight female rats, randomly assigned to ketone ester or standard diet, was measured over two weeks.

All rats were Sprague Dawley CD (Charles River, Wilmington, USA), arrived at 42 days of age, and were put on standard diet immediately after arrival; rats were handled twice over three days; on the morning of the sixth day, rats were either left on standard diet or changed over to ketogenic diet or ketone ester (day 48 of age); rats continued on the experimental diet condition for two weeks (day 62 of age). With the exception of the baseline recordings in the ketone ester condition, all recordings across cohorts were recorded approximately one full day apart. Due to palatability concerns, rats on the ketone ester diet were introduced to the supplement concentration gradually over three days prior to the first full day of ketone ester diet: on day − 3, the standard diet was mixed half-and-half with the ketone ester diet, thus baseline recordings were taken three days prior to start of the conditional diet (day 45 of age). Measurements taken on day 68 of age in the first ketogenic diet cohort were not included: these measurements were taken on different equipment in a different room at different time-of-day. Instead, the measurements taken the afternoon before were considered the final follow-up for the comparison to the second ketogenic diet cohort. Because of this, and in combination with the known animal age variability upon arrival from the distributor, timing characterization should be considered accurate with an error of ±one day.

In order to have a controlled comparison, both the ketogenic diet cohort and the ketone ester cohort were examined in parallel with their own respective standard diet cohorts on the same housing racks at the same time. Ketones, weight, and glucose were compared by two-way repeated measures analysis of covariance (rmANCOVA) between each conditional experimental diet group and its control standard diet group. The overall diet was analyzed in the rmANCOVA by examining the between-subjects effect. The interaction of the diet with time was analyzed within-subjects with use of the Greenhouse-Geisser statistic in the rmANCOVA model. The analysis included sex as a covariate to determine whether the contribution of sex, or if the interaction of sex with diet, impacted the models. Relationships with a significant sex contribution were further analyzed by repeated measures analysis of variance (rmANOVA) between sex in each diet. Measurements from the initial ketogenic diet cohort and the repeated ketogenic diet cohort, which was re-run for confirmation, were compared by rmANCOVA, controlling for sex.

To compare data between the ketogenic diet and the ketone ester groups, effect sizes were assessed using mean values of the variation from each respective controlled standard diet over time. Mean variation and standard deviation values for each day were calculated for each group, producing a Δketones, Δweight, and Δglucose value (and respective standard deviation) for each day. Next, all Δ values and standard deviations were averaged over time. The time-averaged Δ value and standard deviation for ketogenic diet and ketone ester were brought forward for use in Cohen’s d formula. Statistical tests were conducted in SPSS version 24 (IBM, New York, NY) and GraphPad (Prism, San Diego, CA).

## 3 Results

### 3.1 Ketogenic diet compared to standard diet

**Fig. 1:**
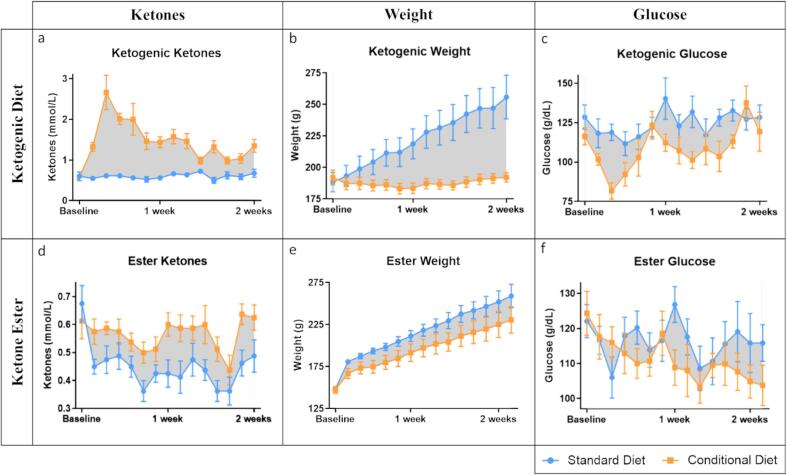
Ketones, weight, and glucose for ketogenic diet and ketone ester conditions graphed alongside their respective standard diet control groups. Data points represent means which include male and female rats; error bars represent standard error; the filled-in gray represents area between the curves.

**Fig. 2:**
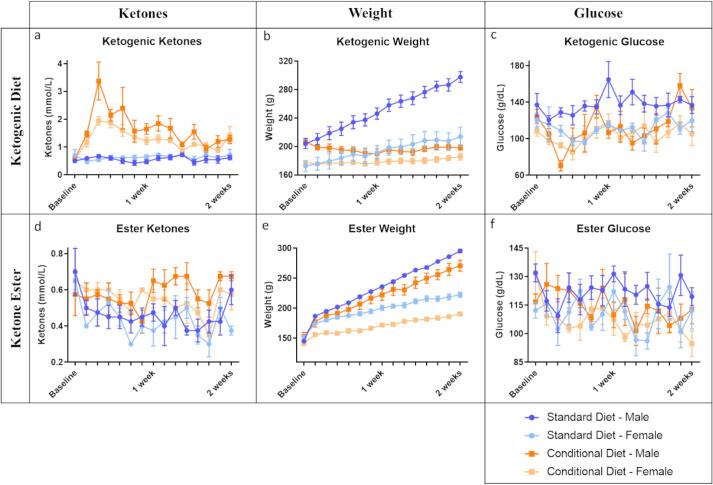
Ketones, weight, and glucose separated out for males and females on the ketogenic diet and ketone ester conditions graphed alongside their respective standard diet control groups. Data points represent means; error bars represent standard error.

As expected, the ketogenic diet condition resulted in increased ketone concentration compared to the standard diet (between subjects F(1,13) = 104.068, p < 0.001; within subjects F(3,42) = 7.199, p < 0.001) (Fig. 1a). The ketogenic diet condition also resulting in an absence of weight gain in contrast to the weight gain observed in the standard diet condition (between subjects F(1,13) = 15.401, p = 0.002; within subjects F(1,18) = 39.383, p < 0.001) (Fig. 1b). In comparison to standard diet, ketogenic diet had an overall decreasing effect on glucose concentration (between subjects F(1,13) = 9.185, p = 0.010), but did not exhibit a consistent lowering effect over time (within subjects F(6,77) = 1.618, p = 0.154) (Fig. 1c). Sex did not interact with the effect of ketogenic diet on ketone concentration (between subjects F(1,13) = 3.123, p = 0.101; within subjects F(3,42) = 1.339, p = 0.274), but did contribute to weight (between subjects F(1,13) = 17.316, p < 0.001; within subjects F(1,18) = 4.317, p = 0.042), and to overall decrease in glucose levels (between subjects F(1,13) = 10.858, p = 0.006) (Fig. 2a–c).

Overall weight differences were observed between sexes in both diet conditions (between subjects SD-F(1,6) = 17.457, p = 0.006 and KD-F(1,6) = 10.365, p = 0.018) with males weighing more than females. Over time, however, weight change did not vary between sexes in the ketogenic diet condition (within subjects F(2,14) = 3.153, p = 0.071). In contrast, weight varied between sexes in the standard diet condition over the course of the study (within subjects F(2,15) = 42.461, p < 0.001). Males began the study weighing 204.2 ± 14.1 g while females weighed 172.6 ± 15.2 g. After two weeks, both males and females on the standard diet consistently increased in weight, with males ending at 297.8 ± 15.1 g and females ending at 214.0 ± 26.7 g (a gain of 31% and 19%, respectively). In contrast, animal weight remained relatively consistent across time in the ketogenic diet condition (ending with a 4% loss in males and 4% gain in females).

Overall glucose sex differences were only observed in the standard diet condition (between subjects F(1,6) = 12.389, p = 0.013) and not the ketogenic diet condition (between subjects F(1,6) = 1.866, p = 0.221). The sex differences in glucose concentration were not detected in either diet condition as a factor of time (within subjects SD-F(4,25) = 1.377, p = 0.270 and KD-F(4,23) = 1.323, p = 0.292). The sex differences in glucose concentration in the standard diet condition, while varying largely (120–165 g/dL in males compared to 96–129 g/dL in females), was represented by a consistently higher concentration in males than females (mean across all days 137.7 ± 10.7 g/dL versus 111.6 ± 9.7 g/dL, respectively). In the ketogenic diet condition, glucose concentration in males more closely resembled females (mean across all days 113.0 ± 20.1 g/dL versus 104.4 ± 9.4 g/dL, respectively).

**Fig. 3:**
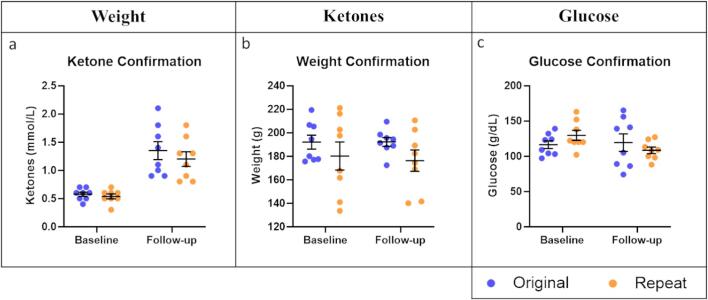
Comparison of original (blue) and confirmation repeat run (orange) of ketogenic diet measurements of a) ketones, b) weight, or c) glucose at baseline (prior to diet changeover) and follow-up (two weeks later). Bars represent mean and standard error. (For interpretation of the references to colour in this figure legend, the reader is referred to the web version of this article.)

In order to confirm that the potential stress of the act of blood sampling was not, in itself, having an effect on circulating ketones and glucose concentration, the ketogenic diet condition was repeated on an identical set of males and females. In this second sampling, measurements were taken only at baseline (prior to initiating the diet condition) and at follow-up (following two weeks of conditional diet) without any sampling between. Ketones, weight, and glucose were compared between the original and repeated sets, controlling for sex, and no significant differences were found (rmANOVA p > 0.05; Fig. 3).

### 3.2 Ketone ester supplement compared to standard diet

The ketone ester condition had an overall effect on increasing ketone concentration (between subjects F(1,13) = 22.610, p < 0.001), but did not exhibit a consistent increasing effect over time (within subjects F(6,79) = 0.930, p = 0.479) (Fig. 1d). The amount of weight that was gained in the ketone ester condition was reduced in comparison to the standard diet condition (between subjects F(1,13) = 19.728, p < 0.001; within subjects F(2,29) = 7.913, p = 0.001) (Fig. 1e). The ketone ester condition did not have an effect on glucose concentration (between subjects F(1,13) = 2.857, p = 0.115; within subject F(7,87) = 0.962, p = 0.462) (Fig. 1f). Sex did not interact with the effect of ketone ester on ketone concentration (between subjects F(1,13) = 2.370, p = 0.148; within subjects F(6,79) = 1.287, p = 0.273), but did contribute to weight (between subjects F(1,13) = 86.795, p < 0.001; within subjects F(2,29) = 74.249, p < 0.001) (Fig. 2d–e).

Weight, and rate of weight gain, varied between sexes in both diet conditions (between subjects SD-F(1,6) = 127.094, p < 0.001 and KE-F(1,6) = 36.088, p < 0.001; within subjects SD-F(3,17) = 105.387, p < 0.001 and KE-F(2,10) = 20.680, p < 0.001), with males weighing more than females, and with males gaining more weight over time.

### 3.3 Ketogenic diet compared to ketone ester supplement

**Fig. 4:**
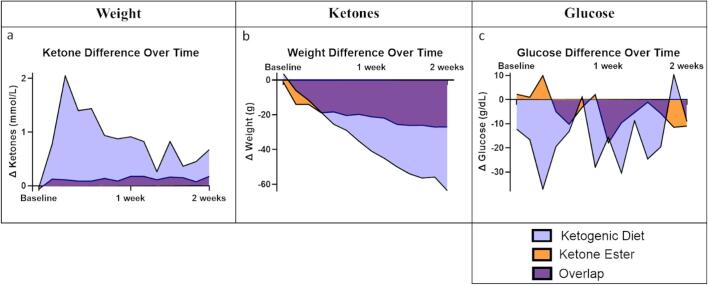
Difference in ketones, weight, or glucose between each conditional diet (KD or KE) and its respective controlled standard diet over two weeks. Both conditional diets are overlaid against the same axis with overlap in purple to contrast between ketogenic diet (blue) and ketone ester (orange). Curves represent means of the conditional diet minus means of the standard diet each day. (For interpretation of the references to colour in this figure legend, the reader is referred to the web version of this article.)

While the ketogenic diet and ketone ester conditions were observed months apart, each conditional arm was conducted with a controlled standard diet condition in parallel, allowing for comparison of the differences from the standard diet. As visualized in Fig. 4, the group mean for ketones, weight, or glucose was calculated each day, and the difference between conditional diet mean and standard diet mean was graphed (Δketones, Δweight, and Δglucose). The daily Δ values and standard deviations were averaged over time to compare the effects that the ketogenic diet and ketone ester conditions had on ketones (KD: 0.84 ± 0.33; KE: 0.11 ± 0.13), weight (KD: −34.9 ± 23.8; KE: −20.1 ± 24.1), and glucose (KD: −16.0 ± 22.2; KE: −4.7 ± 15.8). Cohen’s d for effect size was 2.911 for ketones, 0.618 for weight, and 0.586 for glucose. The ketogenic diet and ketone ester conditions were notably different in glucose and weight (d > 0.5), and the effect that the ketogenic diet condition had on ketones was substantially different from the effect that the ketone ester condition had (greater than two standard deviations).

## 4 Discussion

After two weeks of diet change, both the ketogenic diet condition and the ketone ester condition led to increased circulating ketones and reduced age-related weight gain in normal early-adulthood rats. The ketogenic diet resulted in a greater increase in ketones than the ketone ester did, and the ketogenic diet prevented weight gain while the ketone ester only attenuated it. In addition, the ketone ester had no effect on glucose while the ketogenic diet resulted in reduced glucose. However, this effect was largely driven by higher glucose concentration in male rats on the standard diet which, when on the ketogenic diet, began to approach concentrations more similar to female rats. In addition to glucose, male rats also exhibited higher weight and greater weight gain on the standard diet than females did. On the ketone ester, weight gain rate was reduced across all rats, with a sex interaction that made male rat weight gain rate look more similar to the female rate. On the ketogenic diet, weight gain was prevented entirely, creating a lower boundary in the comparison of male and female weight gain rates in comparison to the standard diet. Overall, while both ketogenic diet and ketone ester conditions have similar effects, the ketogenic diet effects are larger, and male rats are generally influenced by the diet changes to a greater degree than female rats.

The ketogenic diet has been prescribed as a treatment for metabolic diseases, cancers, and seizure disorders (Vidali et al., 2015). However, some have raised questions over the state of health and energy availability while in ketogenesis, particularly in healthy individuals (Malacoff, 2018). Its widespread use over many conditions and circumstances suggests that the ketogenic diet is not harmful (Phinney, 2004), and can even improve exercise performance (Zajac et al., 2014), when followed responsibly for periods of months in adults. Caution should be heeded for developmental periods. The Charles River website for this strain and age range projected that male rats would grow from approximately 200 g to 300 g while female rats would grow from 175 g to 200 g (Materials, 2019). Our standard diet results reflected this expectation: over a two-week period, male rats gained 50% of their body weight and female rats gained 15%. This weight gain was prevented on the ketogenic diet feed. Our staff witnessed rats eating the ketogenic diet feed, and another study found that rats eating this same feed consumed more calories per gram than those on a standard diet (Ma et al., 2018). However, in our project, neither food consumption nor cage activity were recorded, so it is difficult to know if fewer calories were consumed or if more calories were expended. Future behavioral data coming out of our larger project may help to illuminate these questions. Our rats were in early adulthood, but if this phenomenon also occurs in youth, normal development could be affected.

Another concern worth pointing out is the data observed of male rats on the standard diet. Standard diet male rats exhibited blood glucose concentrations consistently over 100 g/dL, frequently falling into the range of 115–140 g/dL. In contrast, glucose concentration of female rats on the standard diet sometimes dipped below 100 g/dL, and often fell into the range of 100–120 g/dL. When placed on the ketogenic diet, glucose concentration in male rats began to resemble glucose concentration in female rats. Notably, patterns of weight, and rate of weight gain (or lack thereof), mirrored the patterns of glucose concentrations between male and female rats across the standard and ketogenic diet conditions. We were informed to expect a rapid weight gain in males on the standard diet, so we considered it normal. However, this raises the question of whether the male rat physiology is, indeed, operating optimally when fed the standard diet. Rats are not humans, but when considering the preponderance of human conditions associated with elevated blood glucose and weight gain, it may be worth examining the physiological response to the standard diet in humans as well, especially in conditions like diabetes.

Due to palatability concerns, some people may want to seek an alternative to the ketogenic diet that produces similar effects. The nuanced results depicted between our ketone ester and ketogenic diet conditions suggests that consumption of ketone esters may not result in the same physiological state as ketogenesis. It could be the case that a dose–response effect exists, and that if we had increased the amount of ketone ester into the feed, it may have produced results more similar to the ketogenic diet. However, if palatability is a concern, it may be increasingly difficult to consume a diet that contains > 20% ketone ester by weight.

There appears to be multiple pathways to achieving ketone metabolism. In the current study, we found that rats on the ketogenic diet had ketone concentrations that peaked at about 3 days, then leveled out at a lower concentration by the 10th day, suggesting that they may have reached a post-hyper-ketogenic adapted state. Had our study gone on longer, and their ketones been increased high enough, and/or for a long enough period of time, perhaps rats on the ketone ester diet would have come to more closely resemble adapted ketogenic diet rats. Alternatively, simply fasting or reducing calories engages ketogenesis (Pilla, 2017). However, it may require regularly fasting for substantial proportions of time (such as one-meal-a-day regiments) in order to increase ketone concentrations high enough, for long enough, to become stable. Similarly, ketogenic-like effects might be attainable by substantially reducing dietary sugar and carbohydrate consumption and/or increasing the proportion of calories from fat in the diet over time. Fat-adaption/keto-adaption diet studies detail metabolic states of apparent glycogen preservation or accelerated restoration and recovery in cyclists (Phinney, Bistrian, Evans, Gervino, & Blackburn, 1983), ultra-endurance runners (Volek et al., 2016), rats (Conlee et al., 1990), race horses and racing dogs (Kronfeld, Ferrante, & Grandjean, 1994), and sled dogs (McKenzie et al., 2005) that are associated with cardiovascular exercise and recovery. In these fat-adaption/keto-adaption studies, diets are proportionally higher in fat and blood ketone levels are increased.

The multitude of means to obtain similar physiological ketone metabolism, or adaption, suggests that the on/off switch for circulating ketones and glucose/glycogen usage may be only part of the story. With the potential for injury mitigation, recovery, and restoration of performance observed in ketone metabolism, military medicine and readiness could be improved by understanding when to harness these mechanisms and under which pathological conditions.

## 5 Disclaimer and Funding Details

The views expressed here are those of the author and do not necessarily reflect the official policy or position of the Department of the Navy, Department of Defense, nor the U.S. Government. This work was supported/funded by Office of Naval Research In-house Laboratory Independent Research work unit number 601152N.0000.000.A1308, and some of the authors were supported by the Naval Research Enterprise Internship Program or the Science and Engineering Apprenticeship Program. The study protocol was reviewed and approved by the Walter Reed Army Institute of Research/Naval Medical Research Center Institutional Animal Care and Use Committee in compliance with all applicable Federal regulations governing the protection of animals in research. The experiments reported herein were conducted in compliance with the Animal Welfare Act and per the principles set forth in the “Guide for Care and Use of Laboratory Animals,” Institute of Laboratory Animals Resources, National Research Council, National Academy Press, 2011. Some of the authors are military Service members or employees of the U.S. Government. This work was prepared as part of their official duties. Title 17, U.S.C., §105 provides that copyright protection under this title is not available for any work of the U.S. Government. Title 17, U.S.C., §101 defines a U.S. Government work as a work prepared by a military Service member or employee of the U.S. Government as part of that person’s official duties. LT Modica and LT Casachahua are both MSC, USN.

## Declaration of Competing Interest

The authors declare that they have no known competing financial interests or personal relationships that could have appeared to influence the work reported in this paper.
